# Assessing the income-related inequality in obesity among the elderly in China: A decomposition analysis

**DOI:** 10.3389/fpubh.2022.918630

**Published:** 2022-09-09

**Authors:** Jinpeng Xu, Guomei Tian, Ting Zhang, Hongyu Zhang, Jian Liu, Qi Shi, Jiale Sun, Haixin Wang, Bokai Zhang, Qunhong Wu, Zheng Kang

**Affiliations:** ^1^Department of Social Medicine, School of Health Management, Harbin Medical University, Harbin, China; ^2^Department of Nuclear Medicine, The Fourth Hospital of Harbin Medical University, Harbin, China; ^3^Central Office, Xuzhou Center for Disease Control and Prevention, Xuzhou, China

**Keywords:** obesity, income-related inequality, concentration index, decomposition analysis, Chinese elderly

## Abstract

**Background:**

Obesity among the elderly imposes a significant health and economic burden. The purpose of this study was to measure the obesity prevalence and income-related inequality among older adults in China and to explore the determinants of the inequity.

**Methods:**

Data were obtained from 4,541 older adults (60 years and older) participating in the China Family Panel Study, 2018. Obesity was defined as body mass index (BMI) ≥28 kg/m^2^. Normalized concentration index and concentration curve were calculated to measure the income-related inequality. Decomposition analysis was used to measure the contribution of each factor to the overall unfairness.

**Results:**

The prevalence of obesity among the respondents was 7.99%. The 95% confidence interval for the overall prevalence was 7.20–8.78%. The normalized concentration index of obesity in the elderly was 0.075 (95% confidence interval: 0.047–0.103), indicating that obesity was more concentrated among the rich (*p* < 0.05). Socioeconomic factors contributed the most to the overall inequality (68.73%). Health behavior factors explained 16.38% of the observed income-related inequality in obesity among the elderly in China.

**Conclusions:**

In 2018, obesity was more concentrated among the elderly with higher incomes in China. The pro-poor income-related inequality was mainly due to the higher socioeconomic status of higher-income older adults. Health behaviors and psychosocial factors could also exacerbate the inequality. To prevent the heavy burden of obesity on the health and finances of older adults, more attention should be paid to those who are financially better off, especially those who smoke and are physically inactive, while extroverted older adults also need to be focused on. For developing countries, concern needs to be given to the obesity of the wealthy elderly as a result of economic development.

## Introduction

In 1997, the World Health Organization declared obesity as a disease (1). Nowadays, obesity has become a global public health problem (2). According to recent data (3), more than 1 billion people worldwide suffer from obesity. The prevalence of obesity is increasing in both developed and developing countries (4–6). The Report on Chinese Residents' Chronic Diseases and Nutrition (2020) showed that more than 50% of Chinese adults are overweight or obese, with the prevalence of obesity being 16.4% (7). Meanwhile, data from the 2020 China National Population Census shows that 18.70% of the Chinese population aged 60 and over (8). China has entered an aging society. The rising prevalence of obesity superimposed on deepening aging, there is a reasonable belief that the obesity prevalence among the elderly in China is on a serious and worrisome increase.

The increasing prevalence of obesity in older adults presents a significant health and economic burden. Obesity increases the risk of various diseases in the elderly (9–12). Cardiovascular diseases, such as hypertension and dyslipidemia, and also some cancers, are more common in obese older adults (13, 14). Overall, obesity is associated with increased mortality risk in the elderly (15). Also, obesity increases health service utilization and health care expenditures for older adults (16). China, as a developing country with the largest elderly population in the world, needs to recognize the challenges posed by obesity in the elderly. Obesity prevention is of great significance in promoting the health status of older adults.

A multitude of social science studies has shown that the occurrence of obesity is by no means a simple biological random event and that people's income levels have a significant impact on obesity in the elderly. However, the direction of this influence varies greatly from country to country. In developed countries, there is a negative correlation between income level and weight, while in developing countries the opposite is observed (17). A study of people aged 50 and over in the UK shows that the poorer elderly are more likely to be obese (18). In Indonesia, obesity is more concentrated in wealthy older adults (19). Philipson and Posner (20), as well as Lakdawalla and Philipson (21), proposed an implied inverted U-shape model in the dynamic framework of weight change. The model states that obese people are relatively wealthier in poor or early societies, but relatively poorer in wealthy and modern societies. Developing countries tend to be in a period of economic development. This period is a critical stage in the “nutritional transition” of people (22). In the last 40 years, the economy of China has grown rapidly. This rapid economic growth has affected the eating habits, lifestyles, and other health behaviors of different socioeconomic classes (23). Studying how obesity is distributed among the elderly of different economic status in China is not only a stage test of China's current economic development, but also can provide empirical support for the future growth of China, in order to address how to achieve the policy goal of “successful aging” in parallel with economic development.

Although several studies have investigated the relationship between socioeconomic status and obesity (24, 25), there is little information about the income-related inequality in obesity among the elderly in China, let alone analyses of the determinants of the inequality. The concentration index has been widely used internationally to measure income-related inequality in the health sector (26), and its decomposition analysis is increasingly being used to examine the determinants of inequality (27). Most previous studies have used concentration index to measure health utilization inequalities, and very limited studies have been conducted on health status. To the best of our knowledge, no studies have been published so far on the income-related inequality in obesity among Chinese older adults (a developing country).

Therefore, the objectives of this study were: (i) to investigate the prevalence of obesity among the Chinese elderly population, (ii) to measure the income-related inequality of obesity among the elderly in China, and (iii) to investigate determinants of the inequality. The results of the study will help identify the most vulnerable older adults with obesity problems in China, shed light on how to reduce income-related obesity inequalities in older age groups in developing countries, and provide targeted recommendations for future policy interventions.

## Methods

### Data source

The rich data from a nationwide survey project, the China Family Panel Study (CFPS) in 2018, provides us with the opportunity to explore the income-related inequality in obesity among the elderly in China. CFPS is a national longitudinal program initiated in 2010 and implemented every 2 years by the Institute of Social Science Survey of Peking University, which aims to investigate families' and individuals' information on a range of topics, including demographic status, economic status, state of health, and so on.

The CFPS baseline survey has six sampling frames, with each of China's five major provinces (Shanghai, Liaoning, etc.) forming five sampling frames and the other 20 provinces together forming one sampling frame (28). Since five large provinces account for a large proportion of the total sample (oversampling), the data from the six sampling frames cannot be directly used for national data analysis, hence the resampled data were used in this study. The CFPS resample data was obtained by resampling the full sample of CFPS data through a probability proportional to size sampling (PPS) procedure. From the technical report provided by CFPS (29), additional weighting is not required for the study using the resampled sample and the sample is representative of the national population. Due to research needs, we selected the elderly aged 60 and above as the participants. After removing cases with missing values, a total of 4,541 samples was obtained.

### Variables

#### Outcome variable

Body mass index (BMI) was used to define the obesity status of respondents. Since the 1990s, BMI has been one of the most common and valid indicators used in obesity research. In terms of international standards, a person is considered overweight when their BMI is higher than 25 and considered obese when their BMI is above 30. However, this classification may not be entirely appropriate for Asians. According to several studies on the obesity problem in China (30), under the support of the International Life Sciences Institute Focal, the Working Group on Obesity in China (WGOC) considers a BMI between 24–28 (excluding 28) as overweight and 28 and above as obese, which is more appropriate for the Chinese (31). Thus, in our study, the respondents were categorized as underweight (<18.5 kg/m^2^), normal weight (18.5 to 23.9 kg/m^2^), overweight (24.0 to 27.9 kg/m^2^), or obesity (≥28.0 kg/m^2^) based on this criterion.

#### Independent variables

Numerous variables are available in CFPS. According to current studies (32–36), after eliminating redundant variables, we considered four dimensions of variables in our study. For multi-category variables, dummy variables were created for all categories, using the highest category as the reference group.

#### Demographic factors

This dimension was composed of gender, age, and marital status. Gender was defined as male and female. Age was categorized into three groups: 60–69 years, 70–79 years, and 80 years and older. Marital status was dichotomized into married or single (including unmarried, divorced, and widowed).

#### Socioeconomic conditions

Socioeconomic conditions included education level, residency, residential economic region, employment status, per capita annual household income, medical insurance, and pension insurance. Education level was categorized into four groups: primary school or below, junior high school, high/secondary school, and college or above. Following the household information of the respondents, the place of residence was divided into rural or urban areas. The economic region was classified according to the National Bureau of Statistics of China into four regions: Northeast, East, Central, and West (37). We used per capita annual household income to measure the income level of older adults and divided individuals based on “rank” for the quintiles, including in the decomposition. Employment status, medical insurance, and pension insurance were both coded as yes = 1 or no = 0.

#### Health behavior variables

Health behavior variables considered in the study were smoking status, drinking alcohol, and physical exercise. As a factor associated with obesity (38), smoking status was measured by asking respondents if they smoked in the past month. Drinking alcohol was measured by whether respondents drank at least 3 times a week in the past month. The information for physical exercise was derived from the question: “How often did you participate in physical exercise in the past week?”

#### Psychosocial factors

The psychosocial factors consisted of personality traits and life satisfaction. Personality traits were measured by using a short 15-item (including four reversed scoring items) version of the Big Five Inventory (BFI-S), which consists of five independent factors, including O (Openness), C (Conscientiousness), E (Extraversion), A (Agreeableness), and N (Neuroticism), each dimension is measured by 3 items. This scale has been used extensively in previous studies (39, 40). It has proven to be reliable and has a sufficient level of utility (41). Referring to Swami, Chamorro-Premuzic (42), we used the mean of the three items for each dimension to measure different personalities, with higher scores meaning a positive tendency on that dimension. To measure respondents' life satisfaction, the participants were asked to rate their life satisfaction with a one-item 5-point Likert scale (ranging from 1, extremely unsatisfied, to 5, extremely satisfied). A score of 3 or more was considered a high-level group, else it was a low-level group.

### Statistical analysis

Stata/SE 16.0 (Stata Corp, College Station, TX, USA) was used for data cleaning and preprocessing. To assess the income-related inequality, the methodology of Wagstaff, Paci (43) was adopted. Concentration index (CI) and concentration curve (CC) were calculated to measure the inequality in obesity. CI values range from −1 to +1. The positive (negative) value indicates that obesity is concentrated among rich (poor) individuals. CI equals zero means there is no inequality (44).


(1) $$\begin{array}{r} CI=\frac{2}{\mu }cov\left(y_{i},r_{i}\right) \end{array}$$

where CI is the concentration index, μ is the mean of obesity, *cov* is the covariance, *y*_*i*_ is the obesity status, *r*_*i*_ is the individual's fractional rank in the economic distribution. As the obesity status is a binary variable, the minimum and maximum of the CI are not −1 and +1. Thus, following Wagstaff's suggestion (45, 46), we normalized the CI as:


(2) $$\begin{array}{r} CI_{normalized}=\frac{CI}{1-\mu } \end{array}$$

Decomposition analysis, first used in some studies in the field of economics and then gradually applied in epidemiology and public health, can be used to determine the contribution of each influencing factor to the unfairness (47, 48). The contribution of each influencing factor is equal to the product of the sensitivity of each factor to the outcome variable and the concentration index of each factor. Since the outcome variables in this study are binary categorical variables, to normalize the decomposition analysis, marginal effects from a probit regression were used in the elasticity calculation (49):


(3) $$\begin{array}{r} y_{i}=\alpha^{m}+\underset{j}{\sum }\beta_{j}^{m}x_{ji}+\varepsilon_{i} \end{array}$$

where *y*_*i*_ is the use of the obesity status by individual *i, x*_*j*_ is a vegetation of independent variables, $\beta_{j}^{m}$ is the partial effects (*dy/dx*_*j*_) for *x*_*j*_, and ε is the error term. The decomposition of the concentration index can thus be expressed as the following formula:


(4) $$CI=\sum (\frac{\beta_{j}^{m}{\bar{x}}_{j}}{\mu })C_{j}+\frac{GC_{\varepsilon }}{\mu }$$

In formula (4), ${\bar{x}}_{j}$ and μ are the mean levels of *x*_*j*_ and *y*_*i*_, respectively. $\left(\frac{\beta_{j}^{m}{\bar{x}}_{j}}{\mu }\right)C_{j}$ is the contributions of independent variables. The negative (positive) contribution of an independent variable indicates that the income-related distribution of this variable and its relation with obesity increases the concentration of obesity among the poor (the rich). $\frac{GC_{\varepsilon }}{\mu }\;$ is the generalized concentration index for the remaining error (50). Applying Wagstaff's correction into formula (4) yields to:


(5) $$CI_{normalized}=\frac{CI}{1-\mu }=\frac{\sum (\frac{\beta_{j}^{m}{\bar{x}}_{j}}{\mu })C_{j}}{1-\mu }+\frac{GC_{\varepsilon }/\mu }{1-\mu }$$

## Results

### Social and demographic characteristics

**Figure 1:**
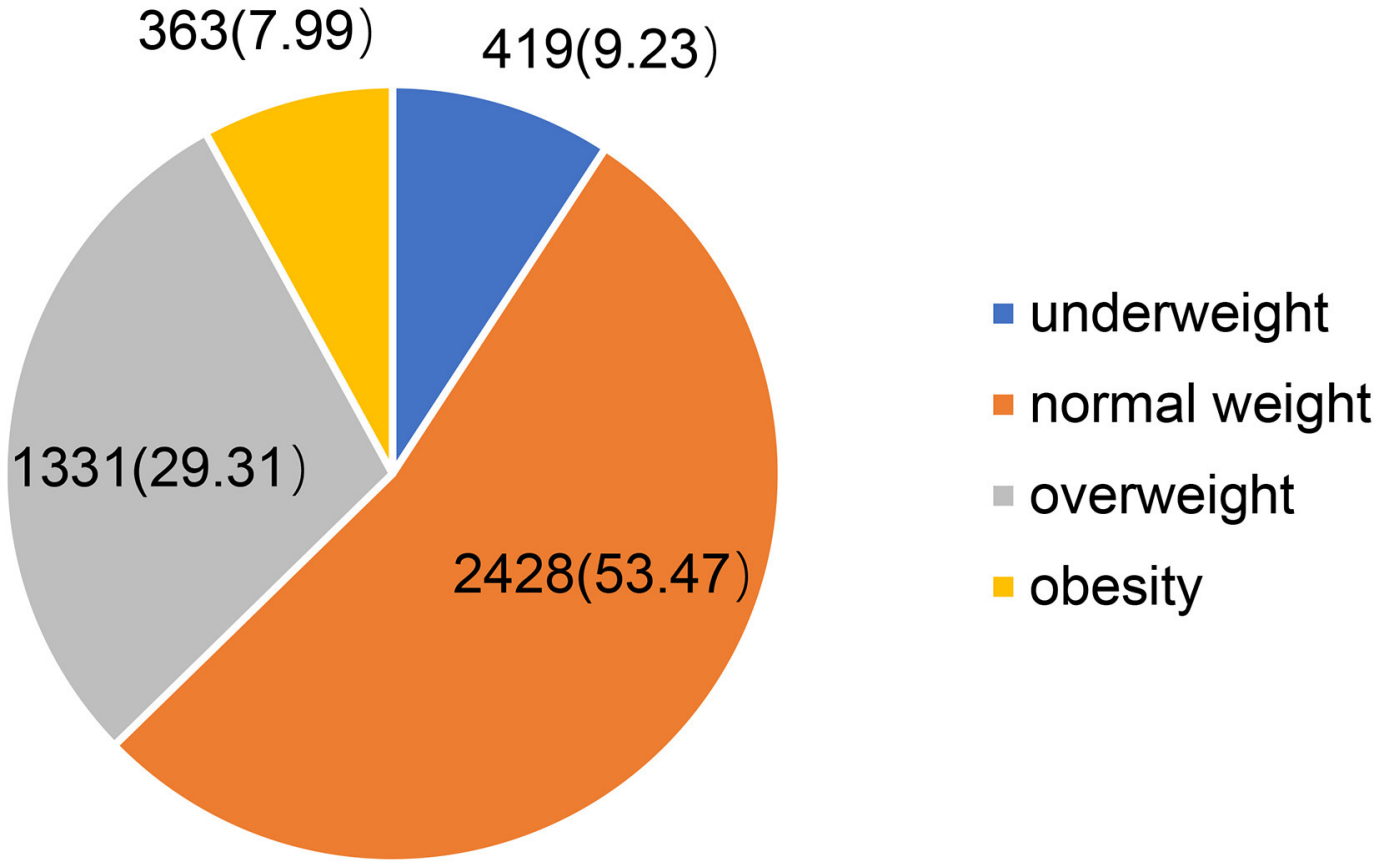
Distribution of BMI of the elderly in China, 2018 (*N* = 4541).

Table 1 shows the characteristics of the respondents. Among the 4,541 older adults, 65.93% were aged 60 to 69 years. There were 2,370 (52.19%) males and 2,171 (47.81%) females. 17.42% of the elderly had no spouse. 69.74% received primary school education or below. In terms of location, 48.87% of the respondents lived in urban areas and 34.99% lived in the eastern part of China. Figure 1 shows the BMI distribution of the interviewed older adults. It can be seen that the prevalence of obesity among Chinese older adults was 7.99%. The 95% confidence interval for the overall prevalence was 7.20–8.78%. Among the three age groups, the highest prevalence was found among the elderly aged 70 to 79 years. With the increase in per capita annual household income, the obesity prevalence of the elderly was also increasing.

**Table 1:** Descriptive statistics for variables (*N* = 4541).

**Characteristics**	***N* (%)**	**Obesity (%)**	
		**No (*n* = 4178)**	**Yes (*n* = 363)**
**Age**			
60–69 years	2994 (65.93)	2751 (91.88)	243 (8.12)
70–79 years	1271 (27.99)	1174 (92.37)	97 (7.63)
80 years and older	276 (6.08)	253 (91.67)	23 (8.33)
**Gender**			
Male	2370 (52.19)	2212 (93.33)	158 (6.67)
Female	2171 (47.81)	1966 (90.56)	205 (9.44)
**Marital status**			
Married	3750 (82.58)	3452 (92.05)	298 (7.95)
Single	791 (17.42)	726 (91.78)	65 (8.22)
**Education level**			
Primary school or below	3167 (69.74)	2912 (91.95)	255 (8.05)
Junior high school	846 (18.63)	782 (92.43)	64 (7.57)
High/secondary school	414 (9.12)	376 (90.82)	38 (9.18)
College or above	114 (2.51)	108 (94.74)	6 (5.26)
**Residency**			
Urban	2219 (48.87)	2016 (90.85)	203 (9.15)
Rural	2322 (51.13)	2162 (93.11)	160 (6.89)
**Residential economic region**			
Northeast China	481 (10.59)	442 (91.89)	39 (8.11)
East China	1589 (34.99)	1438 (90.50)	151 (9.50)
Central China	1285 (28.30)	1173 (91.28)	112 (8.72)
West China	1186 (26.12)	1125 (94.86)	61 (5.14)
**Employment status**			
Yes	2341 (51.55)	2170 (92.70)	171 (7.30)
No	2200 (48.45)	2008 (91.27)	192 (8.73)
**Per capita annual household income**			
Poorest	934 (20.57)	872 (93.36)	62 (6.64)
2nd	941 (20.72)	866 (92.03)	75 (7.97)
Middle	972 (21.40)	897 (92.28)	75 (7.72)
4th	910 (20.04)	835 (91.76)	75 (8.24)
Highest	784 (17.26)	708 (90.31)	76 (9.69)
**Medical insurance**			
Yes	4265 (93.92)	3920 (91.91)	345 (8.09)
No	276 (6.08)	258 (93.48)	18 (6.52)
**Pension insurance**			
Yes	3024 (66.59)	2772 (91.67)	252 (8.33)
No	1517 (33.41)	1406 (92.68)	111 (7.32)
**Smoking status**			
Yes	1320 (29.07)	1247 (94.47)	73 (5.53)
No	3221 (70.93)	2931 (91.00)	290 (9.00)
**Drinking alcohol**			
Yes	870 (19.16)	812 (93.33)	58 (6.67)
No	3671 (80.84)	3366 (91.69)	305 (8.31)
**Physical exercise**			
None	2098 (46.20)	1945 (92.71)	153 (7.29)
1~3 times per week	333 (7.33)	312 (93.69)	21 (6.31)
4 or more times per week	2110 (46.47)	1921 (91.04)	189 (8.96)
Openness, Mean (SD)	3.96 (0.65)	3.96 (0.65)	3.96 (0.72)
Conscientiousness, Mean (SD)	3.45 (0.72)	3.44 (0.72)	3.60 (0.77)
Extraversion, Mean (SD)	3.90 (0.62)	3.89 (0.62)	3.90 (0.63)
Agreeableness, Mean (SD)	3.05 (0.92)	3.05 (0.92)	3.04 (0.95)
Neuroticism, Mean (SD)	2.92 (0.78)	2.92 (0.78)	2.90 (0.77)
**Life satisfaction**			
High	3598 (79.23)	3297 (91.63)	301 (8.37)
Low	943 (20.77)	881 (93.43)	62 (6.57)

### Inequality and decomposition analysis

**Figure 2:**
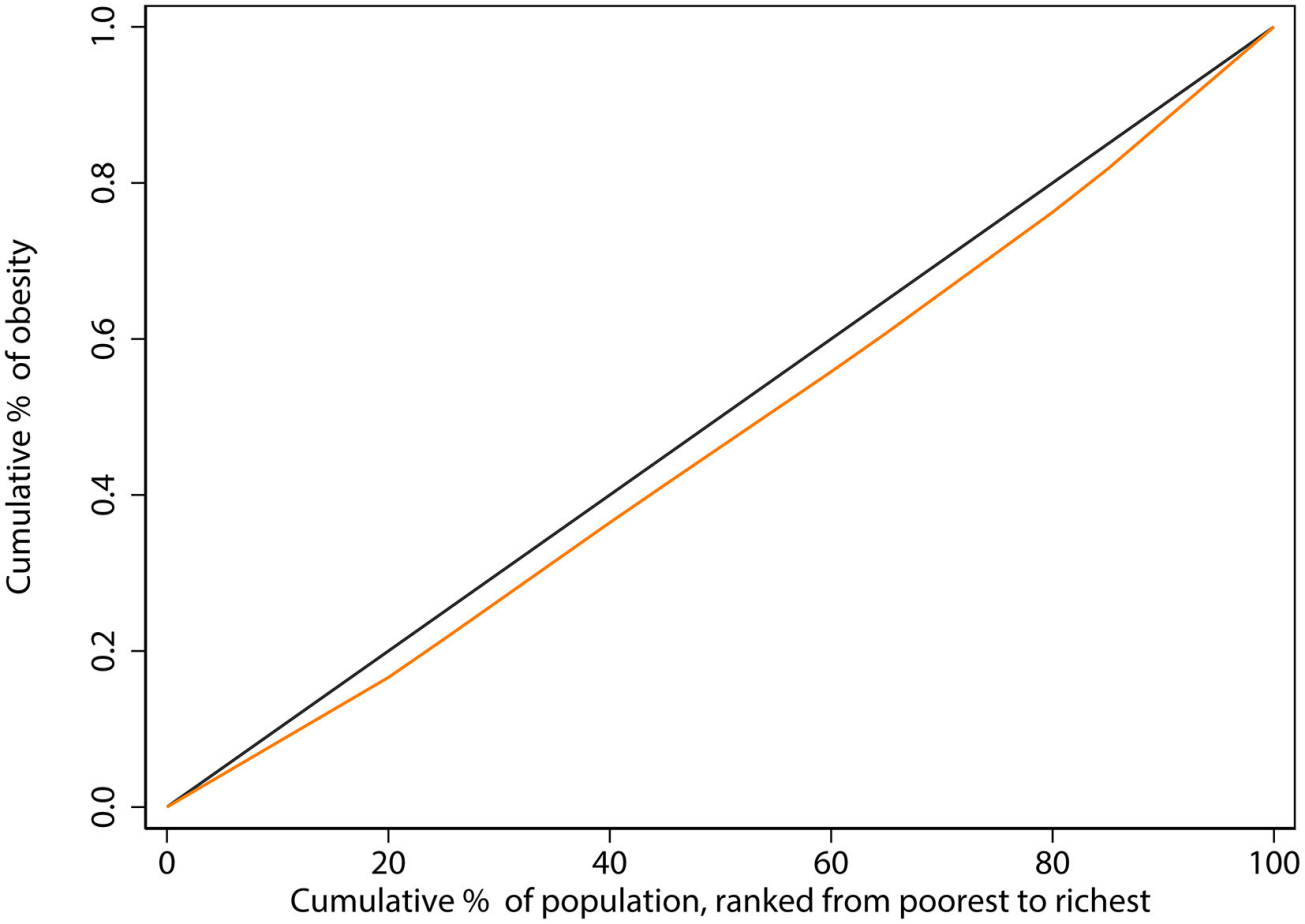
Concentration curve of obesity among the elderly in China. The black line, running from the lower-left corner to the upper-right corner, is the equality line. The orange line below the equality line represents the concentration curve.

Figure 2 displays concentration curves that illustrate the concentration index of the probability of obesity in the elderly. We can observe that the CC is above the equality line. The normalized CI was 0.075, indicating that obesity was concentrated among older adults with higher incomes.

Table 2 shows the results of the CI decomposition analysis. The first column shows the partial effect of each variable on obesity prevalence. The second and third columns indicate the elasticity and concentration indexes of each factor, respectively. The negative concentration index for unhealthy life behaviors, such as smoking (CI = −0.041) and not exercising (CI = −0.128), implies that these behaviors were more concentrated in the lower-income group of older adults. The wealthy group, on the other hand, lived more in urban areas (CI = 0.228) and had higher life satisfaction (CI = 0.017).

**Table 2:** Decomposition of concentration index in obesity among the elderly in China.

**Characteristics**	**dy/dx**	**Elast**	**C_j_**	**Cont to C**	**C%**
**Age**					
60–69 years	−0.002	−0.014	0.020	0.000	−0.402
70–79 years	−0.008	−0.030	−0.059	0.002	2.515
80 years and older	Ref				
**Gender**					
Male	−0.014	−0.088	0.002	0.000	−0.243
Female	Ref				
**Marital status**					
Married	0.002	0.023	0.007	0.000	0.244
Single	Ref				
Subtotal demographic factors				0.001	2.114
**Education level**					
Primary school or below	0.054*	0.467	−0.110	−0.051	−73.886
Junior high school	0.044	0.104	0.153	0.016	22.815
High/secondary school	0.056*	0.063	0.334	0.021	30.542
College or above	Ref				
**Residency**					
Urban	0.017*	0.104	0.228	0.024	34.262
Rural	Ref				
**Residential economic region**					
Northeast China	0.033**	0.043	0.243	0.011	15.183
East China	0.046***	0.203	0.044	0.009	12.830
Central China	0.042***	0.150	−0.075	−0.011	−16.295
West China	Ref				
**Employment status**					
Yes	−0.002	−0.011	−0.136	0.001	2.134
No	Ref				
**Per capita annual household income**					
Poorest	−0.018	−0.045	−0.794	0.036	51.845
2nd	−0.004	−0.011	−0.381	0.004	5.949
Middle	−0.011	−0.029	0.040	−0.001	−1.638
4th	−0.008	−0.020	0.454	−0.009	−13.153
Highest	Ref				
**Medical insurance**					
Yes	0.011	0.133	−0.001	0.000	−0.137
No	Ref				
**Pension insurance**					
Yes	0.010	0.083	−0.014	−0.001	−1.725
No	Ref				
Subtotal socioeconomic conditions				0.048	68.727
**Smoking status**					
Yes	−0.025**	−0.092	−0.041	0.004	5.397
No	Ref				
**Drinking alcohol**					
Yes	−0.003	−0.007	0.013	0.000	−0.135
No	Ref				
**Physical exercise**					
None	−0.012	−0.067	−0.128	0.009	12.335
1~3 times per week	−0.021	−0.019	0.044	−0.001	−1.215
4 or more times per week	Ref				
Subtotal health behavior variables				0.011	16.382
Openness	0.025	−0.335	0.008	−0.003	−3.616
Conscientiousness	−0.007	1.059	0.004	0.004	6.050
Extraversion	−0.001***	−0.291	0.010	−0.003	−4.185
Agreeableness	−0.006	−0.046	0.003	0.000	−0.217
Neuroticism	−0.001	−0.049	−0.018	0.001	1.289
**Life satisfaction**					
High	0.012	0.116	0.017	0.002	2.863
Low	Ref				
Subtotal psychosocial factors				0.002	2.183
Residual (unexplained)				−0.005	10.594

dy/dx, Marginal effects; Elast, elasticity; C_j_, Concentration index of explanatory variables; Cont to C, Contribution to the overall concentration index; **C%**, Percentage contribution to CI; Ref, reference group.

P–value, *p < 0.10; **0.05 ≤ p < 0.10; ***p < 0.01.

The last two columns show the absolute contribution of each factor to the overall concentration index and the absolute percentage contribution of each factor. Decomposition analysis showed that having a primary school education or below and living in the central region contributed negatively to the CI of obesity among the elderly, reducing the income-related inequity of obesity among the elderly, while higher education, living in urban areas, smoking, not exercising, being extroverted made positive contributions, suggesting that obesity inequality would be lower in the absence of these factors.

**Figure 3:**
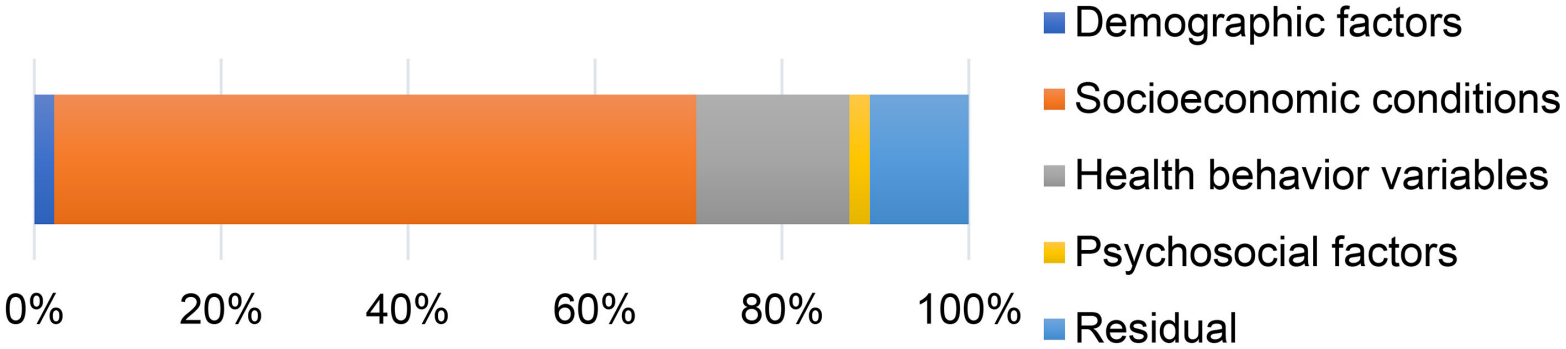
Contribution to inequality in obesity among the elderly in China (%).

Finally, we analyzed how the variables of different dimensions contribute to the observed income-related inequality in obesity among older adults. The results are shown in Figure 3. We found that all factors contributed positively to the inequality. Socioeconomic factors explained a large part of the inequality in obesity with a contribution of 68.73%, followed by health behaviors with a contribution of 16.38%.

## Discussion

Obesity is a major risk factor for non-communicable diseases (51) and can place a severe physical and economic burden on older adults. Reducing obesity can help to significantly decrease the catastrophic health effects of diseases arising from obesity, thereby reducing mortality and disability-adjusted life years lost (52). This is the first study to assess the prevalence of obesity and its income-related inequality of obesity among older adults in China with a detailed decomposition analysis of the concentration index, which can help identify effective ways to improve this obesity inequity. In addition to this, we used nationally representative survey data from the CFPS. The findings are applicable to a broader population in China and may provide more compelling evidence for research on obesity among older adults in developing countries.

More than half of the older adults included in this study were at normal weight levels, and 29.31% were overweight. The prevalence of obesity among the elderly was 7.99%, a value similar to that of the Chinese labor force (53), but lower than the results from Iran (54) and Malaysia (55). Compared to other developing countries in Asia, our study observed a lower prevalence of obesity among the elderly. Nevertheless, income-related inequality in obesity was identified. In this study, we found a positive concentration index of obesity among Chinese older adults. This indicates that obesity was concentrated among older adults with higher incomes. Most results in developing countries show a positive association between socioeconomic status and obesity prevalence, that is, the higher the socioeconomic status of the group, the higher the prevalence of obesity (56, 57), while some studies from developed countries show the opposite (18, 58). Similar to results from both developed and developing countries (59–61), socioeconomic status, region of residence, physical activity, and smoking were the main positive factors contributing to income-related inequalities in obesity. This phenomenon can be explained in several aspects.

Regarding the socioeconomic situation of the elderly, we found that having an education level of primary school or below contributed −73.89% of the income-related inequity, weakening the pro-poor inequality of obesity. This is mainly because less-educated older adults tend to be concentrated in the low-income group, who have a lower health awareness and health literacy (62) and are not concerned about their health behaviors such as a balanced diet and exercise. At the same time, observing the concentration index of each factor, it can be found that in China, high-income elderly people tended to live in urban, eastern China, these areas have a higher level of economic development. The higher intake of high-fat, high-sugar foods in the diets of older adults make them more prone to obesity problems, contributing to the pro-poor inequality.

From the perspective of health behaviors of the elderly, we found that the prevalence of obesity was lower among elderly smokers than non-smokers, which is consistent with the findings of previous studies (63). This may be related to the fact that smoking increases the body's energy expenditure and suppresses appetite (64, 65). Jessen, Buemann (66) showed that if the dose of nicotine in cigarettes was gradually increased over 2 h, hunger and food consumption were negatively correlated with their dose, while satiety was positive, all of which contributed to lighter weight in older adults who smoked. Physical exercise improves muscle loss and fat loss in the elderly (67), and regular physical activity can prevent the decline of BMI to lower levels in older adults. For subjective reasons like low health awareness or objective reasons like lack of exercise equipment, the poor tend not to exercise compared to older people with higher incomes, so the contribution of not exercising to the overall obesity inequity was positive.

To our knowledge, most current research tends to explain how a person's BMI affects their psychosocial status, such as depression (68), subjective well-being (69), life satisfaction (70), etc., with little academic attention in the opposite direction. Therefore, in this study, we included variables such as personality traits and life satisfaction to measure the effect of psychosocial factors on obesity in older adults. It proved that our approach is valuable. Older adults with extroverted personalities were more likely to be obese. Button, Faith (71) found that children with more extroverted and impulsive personalities eat more quickly and may be more prone to obesity than children who eat more slowly. Our study confirms the applicability of this finding to an older population. At the same time, older adults who were satisfied with their lives had a higher probability of being obese, consistent with the findings of several previous studies (72, 73). This suggests that there is a pattern of “happy eating” among the elderly in China, where the happier the elderly feel, the more they will eat. Although the underlying biological and neural mechanisms have not been fully identified, the phenomenon is widespread in different groups and countries (74). Therefore, more attention needs to be paid to how to ensure that older people have a happy and healthy life in their old age.

According to the decomposition results of the concentration index, socioeconomic factors contributed the most to the income-related inequity. All other factors contributed much less to the concentration index than socioeconomic factors such as the economic region of the elderly and the annual per capita household income, and even though they also increased the obesity inequity of the Chinese elderly to some extent, the gap among them was still very obvious. This in turn suggests that the income-related inequity among the elderly in China was mainly due to the higher socioeconomic status of older adults with higher incomes. In developing countries, along with the growth of wealth and food supply, obesity is more of a “disease of the rich” among high socioeconomic status groups. For developing countries, economic development should be accompanied by attention to the obesity problem of the wealthy elderly.

These findings provide some new inspiration for the intervention of obesity in older adults. Firstly, the determinants of income-related inequity of obesity among the elderly were mainly socio-economic factors, which suggests that the elderly with better economic conditions are the current priority group for obesity intervention and control. Secondly, for the low-income group, we should promote them to form good life behavior habits such as exercise through policy promotion. And thirdly, considering that health behaviors and psychosocial factors could also exacerbate the inequality of obesity among the elderly, we need to focus on seniors who smoke, don't exercise, and are extroverted.

Despite the above findings and recommendations, several limitations of this study need to be acknowledged. The most important limitation is that, due to restrictions in questionnaire design and survey methodology, our only measure of obesity is BMI, so we cannot directly measure aspects of body composition, such as visceral fat or fat distribution of the elderly (75). In addition, our study used a cross-sectional design, but the effect of each factor on individual obesity is long-term, making follow-up data more appropriate than cross-sectional data for analyzing this effect over time. Finally, because all data used in our study were self-reported, reporting bias and recall bias were inevitable despite some quality control measures in place.

## Conclusion

Overall, this study examined the prevalence of obesity and income-related inequality among the elderly in China. In a developing country like China with an increasingly aging population, our study has important implications. The study shows that obesity was more concentrated among older adults with higher income in China in 2018. Socioeconomic factors, health behaviors, and psychosocial factors all contributed to the pro-poor inequality. Located in economically developed areas, smoking, lack of exercise, and extroversion all contribute in varying degrees to the obesity of older adults. This income-related inequality was mainly caused by the fact that older adults with higher income have higher socioeconomic status. To prevent the severe health and economic burden caused by obesity in the elderly, we should pay more attention to older adults who are better off, smoke, don't exercise, and are extroverted. In developing countries, obesity among wealthy older adults caused by economic development should be a concern.

## Data availability statement

The datasets presented in this study can be found in online repositories. The names of the repository/repositories and accession number(s) can be found below: http://www.isss.pku.edu.cn/cfps/en/index.htm.

## Author contributions

JX conceived the idea and wrote the manuscript. GT and TZ designed this study and conducted statistical analyses. ZK contributed to the writing-review and editing and funding acquisition. HZ, JL, QS, and JS acquired the data and contributed additional advice regarding the analysis. BZ and HW provided constructive suggestions for the discussion of the manuscript. GT and QW critically revised the paper. All authors read and approved the final manuscript.

## Funding

The research was supported by: (1) National Natural Science Foundation of China (72074064 and 71573068). (2) China Postdoctoral Science Foundation (2019M650068 and 2018T110319). (3) National Social Science Foundation of China (19AZD013).

## Conflict of interest

The authors declare that the research was conducted in the absence of any commercial or financial relationships that could be construed as a potential conflict of interest.

## Publisher's note

All claims expressed in this article are solely those of the authors and do not necessarily represent those of their affiliated organizations, or those of the publisher, the editors and the reviewers. Any product that may be evaluated in this article, or claim that may be made by its manufacturer, is not guaranteed or endorsed by the publisher.

## Abbreviations

CFPS: China Family Panel Study
BMI: Body mass index
CI: Concentration Index
CC: Concentration Curve.
